# Abrupt height growth setbacks show overbrowsing of tree saplings, which can be reduced by raising deer harvest

**DOI:** 10.1038/s41598-023-38951-8

**Published:** 2023-07-25

**Authors:** Kai Bödeker, Claudia Jordan-Fragstein, Torsten Vor, Christian Ammer, Thomas Knoke

**Affiliations:** 1 Institute of Forest Management, Department of Life Science Systems, TUM School of Life Sciences Weihenstephan, Technical University of Munich, Hans-Carl-von-Carlowitz-Platz 2, 85354 Freising, Germany; 2 Chair of Forest Protection, Institute of Silviculture and Forest Protection, Tecnical Universeity of Dresden, Pienner Straße 8, 01737 Tharandt, Germany; 3 Faculty of Resource Management, University of Applied Sciences and Arts, Büsgenweg 1a, 37077 Göttingen, Germany; 4 Silviculture and Forest Ecology of the Temperate Zones and Centre for Biodiversity and Sustainable Land Use, University of Göttingen, Büsgenweg 1, 37077 Göttingen, Germany

**Keywords:** Biodiversity, Forest ecology, Forestry

## Abstract

Intensive ungulate browsing significantly impacts forests worldwide. However, it is usually not single browsing events that lead to sapling mortality, but the little-researched interactions of browsed saplings with their biotic and abiotic environment. (I) Our objective was to assess the impact of ungulate browsing on the growth of young saplings relative to other environmental factors by utilizing their height increment as a sensitive measure of vitality to indicate their status. (II) Furthermore, we aimed to identify factors affecting ungulate browsing at our study sites, assessed as browsing probabilities, and identify effective mitigation measures for browsing impact. We analyzed an extensive sapling dataset of 248 wildlife exclosures, which were erected in 2016 in beech dominated forests across Germany and assessed annually until 2020. (I) Browsing probability and light availability were the most influential parameters for selectively browsed, admixed tree species (e.g., sycamore maple). Height increment showed abrupt setbacks, which caused a permanent collapse of growth when browsing exceeded a certain level. However, light availability enhanced height increment. (II) An increase in deer harvest reduced the browsing probability of selectively browsed species considerably. We conclude that the growth-inhibiting effect of ungulate browsing is a multifactorial phenomenon, which can be mitigated by silvicultural management and efficient hunting strategies.

## Introduction

In Europe, browsing by ungulates like roe (*Capreolus capreolus* L.) and red (*Cervus elaphus* L.) deer is a key variable shaping forest structure, composition, and development^1–3^. Long before such large-scale changes become visible, the effects of browsing on trees can be observed on the individual sapling level^4,5^.

Successive browsing on the primary shoot can significantly reduce sapling height growth, a crucial indicator of plant vitality and mortality risk^4,6,7^. As plants compete for scarce resources such as light, browsed saplings are at a distinct disadvantage compared to untouched ones due to factors like ungulate preferences or location^8–12^. The legacy of this competitive disadvantage can persist long after affected saplings outgrow ungulates’ maximum browsing height and can result in their mortality^13^. Consequently, selectively browsed species are typically underrepresented in mature mixed stands, rendering those stands less diverse^4,12,14^. This is important because stand species diversity can promote productivity^15,16^, mitigate the risk of stand failure following natural disturbance^17–19^ and foster elevated biodiversity (e.g., the floristic diversity)^20,21^—key considerations for climate-smart multifunctional forestry^22,23^. Beyond that, there are compelling instances in the scientific literature illustrating that ungulate browsing can precipitate extensive regeneration failures, or prevent forests from achieving their mature state^14,24^.

However, ungulates and forest owners tend to favor the same tree species that are relatively scarce. Consequently, to promote the growth of desirable tree species, forest owners may need to employ expensive methods such as fencing to reduce browsing pressure^6,17,25^. For managers to cost-efficiently promote these selectively browsed species in mixed stands, more information is needed regarding the major factors influencing their height growth, browsing intensity thresholds, and the efficacy of common browsing-control measures like hunting.

At a physiological level, both height growth and browsing resilience are predictably influenced by abiotic factors like soil acidity, water and nutrients^26–28^. In a terminal shoot clipping experiment, Csilléry et al.^11^ conclude that “browsed” silver fir (*Abies alba* L.) saplings can recover quicker on rich soils. Furthermore, light availability on the forest floor is a central resource influencing the vitality, morphology, and radial and height growth of regenerating forests^4,29–31^. However, our current understanding of the significance of these growth-influencing parameters largely relies on case studies centered on economically important species like beech, Norway spruce (*Picea abies* L.), and silver fir (*Abies ablba* L.) (e.g., Csilléry et al.^11^ and Kupferschmid et al.^29^). Surprisingly, research has largely overlooked less common species like rowan (*Sorbus aucuparia* L.), birch (*Betual spp.*), and sycamore maple (*Acer pseudoplatanus* L.)—even though these species are rare, in part, because they are selectively browsed by ungulates, making them potentially sensitive indicators of changing browsing patterns^32^. Even if more data about the physiological responses of these species to browsing were available, the existence of ecological feedbacks would make it difficult to extrapolate to the stand or landscape scale. For instance, greater light availability supports not only the growth of saplings, but also of other understory species like shrubs and herbaceous plants^33,34^. More understory biomass usually means better forage, which may help explain why browsing event surges tend to track with increasing canopy gap size^34,35^. In such cases, it is not only important for forest managers to know how individual saplings may respond to browsing damage, or how to mitigate damages; guidance on browsing itself is also needed.

Efforts to generate evidence-based guidelines for acceptable browsing thresholds represent a first step in this direction. Eiberle and Nigg^7,36^, for instance, used the correlation between relative height increment loss and mortality to suggest browsing thresholds which, if exceeded in the long term, could threaten the viability of the browsed tree species. However, these thresholds are based on relatively coarse assumptions (e.g., sapling mortality occurs if height growth decreases by more than 25%), have a very limited local validity (Swiss mountain forests), and neglect all growth parameters apart from browsing. Nonetheless, these thresholds are, to the best of our knowledge, the only ones of their kind and are used in the field to size up ungulate browsing damage in European forests^13,32,37^.

How should forest managers act when when overbrowsing has become problematic? In Central Europe, providing excellent ungulate habitat conditions and where large predators are either rare or absent^38,39^, ungulate numbers have increased steadily over the last century^40,41^. In the absence of predators, controlling ungulate populations and browsing behavior via hunting is the most common means of regulating browsing impacts^42,43^. Scientists are divided, however, about whether its effectiveness is borne out by the empirical evidence. In Canada, for instance, significant increases in ungulate cullings failed to generate a measurable effect on browsing impact^44^, and hunting-induced reductions in ungulate density in the Czech Republic failed to protect rare, selectively browsed species^45^. Hothorn and Müller^46^ present contradictory evidence that hunting can, in fact, control browsing impact—but only if the ungulate harvest sizes suggested by authorities are based on regularly-conducted browsing damage surveys. Possible rationales for their observation might be a downsized ungulate population, or fear triggered by hunting, leading to avoidance of the regeneration sites^43^.

This study aims to contribute to a more robust understanding of dynamics at the ungulate-sapling-environment nexus, with a special emphasis on selectively browsed European tree species. To that end, we present a detailed analysis of results from a large-scale ungulate exclusion experiment designed to capture regeneration dynamics in versatile forests across Germany, featuring 248 study sites spanning a wide range of browsing intensities, sapling densities, growth conditions, and hunting regimes, based on the following research questions:

(I) What is the influence of ungulate browsing on selectively browsed, admixed saplings’ height growth compared to other environmental factors?
(II) How much deer browsing is tolerable on our study sites without risking unrecoverable height growth losses?
(III) Can local hunting activities alter ungulate browsing impact?

## Methods

### Study sites and experimental design

**Figure 1:**
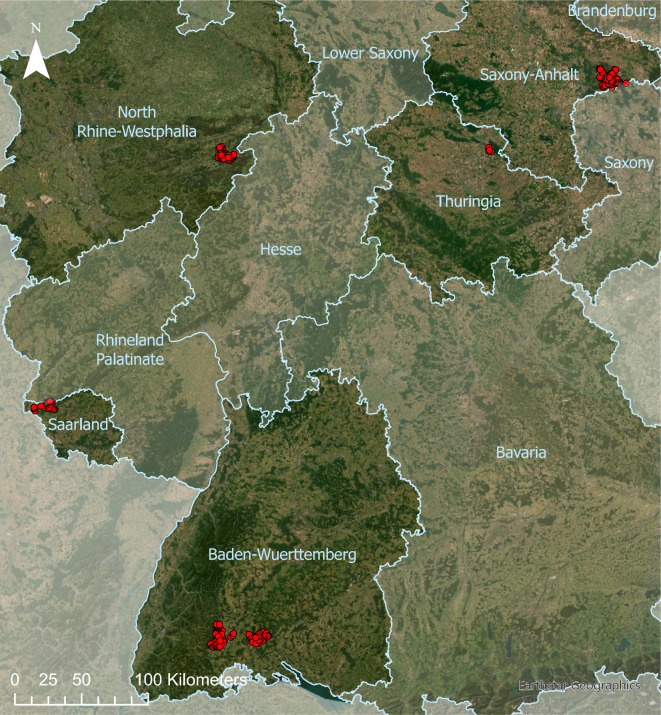
Map of the designated monitoring areas, each marked with a red point, covering five German sates.

The data we used in this research was captured through the BioWild project. Forest regeneration data was gathered in five different German states (Baden-Wuerttemberg, North Rhine-Westphalia, Saarland, Saxony-Anhalt, and Thuringia) (see Fig. 1). Throughout the states, 248 separate 100 m^2^ (10 × 10 m) large wildlife exclosures were established in 2016, with each one having an identical control plot (unfenced, with ungulate impact). Each monitoring area (a single exclosure plot with the associated reference plot) was arranged with site conditions as similar as possible (light, soil, topography, regeneration emergence). The fencing enclosed an area of 144 m^2^ (12 × 12 m) to build a one-meter browsing buffer zone around the actual plot^47^. The pair of plots were carefully laid out to ensure they did not directly adjoin each other, to avoid atypical site conditions as wildlife tend to travel along fences^48,49^. Applying the statistical model by Kolo et al.^50^, the monitoring areas were selected based on the highest probability of regeneration occurrence. Every monitoring area represented maximally 100 ha of forest.

### Vegetation surveys

Between 2016 and 2020 annual vegetation surveys took place. The year 2016 represents the initial state. To guarantee a uniform dataset of high quality, the vegetation data was captured by the same team each year, using the same acquisition protocol in late summer. Beech was the dominant species. For woody vegetation between 50 and 500 cm, the species was identified, height was measured, and their terminal shoots were checked for browsing [True/False]. Though height was measured annually, the individual trees were not tagged and tracked over time. We used the total sapling density per plot, regardless of species, to include the impact of potential inter- and intraspecific competition.

### Environmental variables

Several variables of potential impact on the growth development of forest regeneration were acquired (see Supplementary Table S1):

#### Light variables

Annual light conditions were captured for each plot using hemispheric images produced via a fisheye lens device (Solariscope, Behling SOL300) to compare with Annighöfer et al.^30^ . We observed the direct (DSF), indirect (ISF) and total site factor (TSF) measurements. The light measurements were performed at 2 m above the ground with 5 replications.

#### Hunting

Prior to the project starting, the representatives of administrative hunting districts were asked to choose between two hunting regimes. In regime A, the aim was to reduce browsing pressure through hunting. To achieve that objective, hunters were instructed to shoot any ungulates they encountered that could legally be hunted. Also, hunters and forest owners were asked to cooperate closely with each other, to communicate their goals. Ungulates should be disturbed as little as possible by limiting the frequency of hunting activities. However, when hunting was executed, it should have been in the form of more intense, coordinated efforts such as driven hunts, rather than individuals hunting alone. The intentions of hunting regime A were not subject to any implementation control. Regime B provided the option to spare animals because of hunting customs (such as male deer without antlers). Hunting was carried out as before the start of the project. Both hunting regimes (A and B) were carried out by sport hunters. Given the subjective characteristics of the hunting regime variable, it was solely utilized as a stratifying variable in the context of question III.

Hunting information is quantified by roe deer kills per 100 ha^−1^ a^−1^ for every hunting district containing a monitoring area. Roe deer were shot in every hunting district. We provided an additional globally summarizing ungulate harvest category, as in some districts red deer, fallow deer (*Dama dama* L.) and wild boar (*Sus scrofa* L.) shots were also reported. No hunting district was populated by wolves (*Canis lupus* L.), though sporadic wolf sightings were made in Saxony-Anhalt in the later phase of the project. We attempted to rate local hunting efficiency by providing the shots of roe deer and ungulates per 100 ha per month in which people were actively hunting.

#### Additional Variables

We complemented our field data with additional variables potentially impacting growth and browsing, derived from publicly available sources (see Supplementary Table S1). Annual weather data from the German Climate Data Center were joined to our data, including mean air temperature 2 m above ground, quantity of days with a minimum temperature below 0 °C and maximum temperature above 30 °C, precipitation, sunshine duration, day the vegetation growth period began and ended. All weather maps came preprocessed and resolve with a 1 km grid; the weather data processing was conducted by the German Climate Data Center according to the “General Meteorological Standards”^51–53^. Soil texture and soil type information was joined to our dataset by utilizing soil survey maps (scale 1:1,000,000) by the German Federal Institute for Geosciences and Natural Resources^54^. We incorporated fragmentation and forest edge effects by calculating the forest size and forest edge circumference in which the monitoring areas were located. Also, adapted from Takarabe and Iijima 2020^55^, we calculated the forest proportion in a 750 m radius around the center of each monitoring area and the distance of the center to the closest forest edge. To account for local terrain or relief effects, we first determined the elevation with a 38.2 m resolution at a 0° latitude, using the R-Package elevatr^56^. Based on the created elevation raster-data we could compute the slope, aspect, topographic position index (TPI), terrain ruggedness index (TRI) and roughness according to Wilson et al.^57^ using the raster R-Package^58^.

### Data preparation

As individual saplings could not be tracked over time, we could not directly distinguish in- or out-growing saplings into the 50–500 cm height class, or trees that recently died off. To focus on the height increment of the original saplings from 2016, we constructed an extensive filter function. The function retrieves its first filter conditions from the 2016 data separately for every species and plot, by counting the individuals and determining the smallest sapling size. Applying these plot-species-specific conditions to the following year’s data, we created subsets of all trees larger than the smallest of the condition-year. If the subset then had a higher sapling count than in the previous year, only the tallest individuals were selected. If the subset had fewer individuals than the year before, the number of missing saplings was collected in a separate missing-saplings dataset. If no information was found for a filter condition, the individuals of the previous year were also collected in the missing-saplings dataset. If the height of the saplings absent from the previous year’s data was less than 400 cm, the algorithm categorized them as “no longer within our observation scope” (potentially due to mortality). Conversely, if the saplings’ height exceeded 400 cm, they were considered as potentially having grown beyond our measurement range. After the filter function ran through all plots and species of the subsequent year, the new dataset formed new filter conditions. During the filtering process, 2016 data stayed untouched, as it initialized the starting condition. In the end, we obtained two datasets: one documenting the development of the live saplings since 2016, and one recording the saplings that either died (for an indeterminate reason) or grew out of the height class. The count of the potentially dead saplings was later appended to the live sapling dataset as an additional explanatory variable. We determined annual height growth (for question I and II) as the difference between the median heights of consecutive years per species group and plot.

Due to the focus of this study on rare, preferentially browsed species, we increased the sample size for our models (except for the height growth filter function) by grouping rare species with similar properties according to the German National Forest Inventory (compare Lorenz et al.^59^). We formed 3 tree species groups (see Supplementary Table S2):

- “Other deciduous tree species with a high life expectancy” (ODH) (main tree species: sycamore maple (*Acer pseudoplatanus* L.))
- “Other deciduous tree species with a short life expectancy” (ODS) (main tree species: (*Sorbus aucuparia* L.))
- Selectively browsed conifers (main tree species: silver fir).

### Data analysis

We parsed our data using a two-step approach. First, we computed our dependent variables (height growth for questions I and II, and browsing for question III), which we then predicted using a random forest model. Random forest models are a type of ensemble learning technique that builds multiple decision trees^60^. We chose this approach because random forests are exceptionally well-equipped to evaluate the predictive influence of numerous independent variables on dependent variables, regardless of their statistical distribution^61,62^.

We quantified ungulate impact by predicting the annual browsing probability (BP) for every plot and species using a logistic mixed effect regression based on the data of every state (compare to^32,63^). The mixed effect logistic regression accounts for the binary nature of our browsing response variable [true/false] and allows predictions for plots where a small sample size would result in extreme stochastic browsing percentages^32,62^. Furthermore, ungulate browsing is known to be spatially and temporally correlated^64^, which we addressed by implementing the random effect. We applied a fixed nested effect between data acquisition year and species group (without intercept) and let a random intercept vary among hunting district and plot with a random slope on year (see Eq. 1). Following the approach of Hothorn et al.^63^, we enforced that the linear approximation passes through the origin to obtain expected values for each fixed categorical predictor. BP was predicted for saplings in roe deer reach (<1.30 m) using the fixed and random effect structure of the model.

1 $$\begin{aligned} \eta _{h,p} = \beta _1(\text {year}_{h,p} \times \text {species.group}_{h,p}) + \beta _2\text {year}_{p,h} + \gamma _{2,h}\text {year}_{h,p} + \gamma _{2,p \mid h}\text {year}_{h,p} + \gamma _{0,h} + \gamma _{0,p \mid h} + e_{h,p} \end{aligned}$$

where:

$$\begin{aligned} \eta _{h,p}&= \text {linear predictor for the browsing probability,} \\ \beta _1&= \text {fixed slope parameter for the interaction of year and species.group,} \\ \text {year}_{h,p} \times \text {species.group}_{h,p}&= \text {interaction term,} \\ \beta _2\text {year}_{p,h}&= \text {fixed slope parameter for year,} \\ \gamma _{2,h}\text {year}_{h,p} + \gamma _{2,p \mid h}\text {year}_{h,p}&= \text {random slope for year, varying by hunting district } h \\&\quad \text {and plot } p \text { nested within hunting district } h, \\ \gamma _{0,h} + \gamma _{0,p \mid h}&= \text {cluster-specific (random) deviations from the intercept, varying by hunting district } h \\&\quad \text {and plot } p \text { nested within hunting district } h, \\ e_{h,p}&= \text {residual error terms.} \end{aligned}$$

We then used BP in the random forest regression model as an explanatory variable for research questions I and II to observe the influence of browsing on the “early mortality detection measure” height growth. For question III we used BP as the dependent variable, given its direct correlation with ungulate height growth loss.

**Figure 2:**
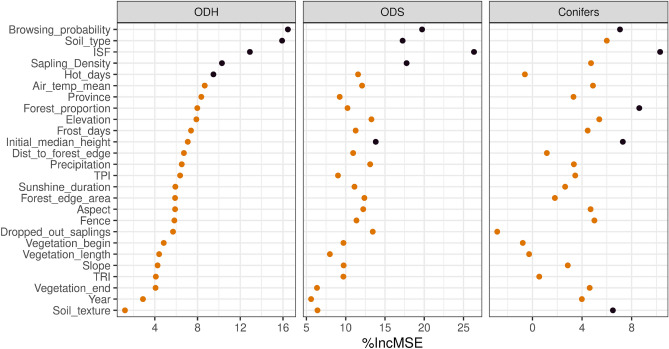
Permutation importance (relative increasing mean square error) predicting height growth for all explanatory variables and the tree species groups ODH (other deciduous tree species, high life expectancy), ODS (other deciduous tree species, short life expectancy) and conifers. The explanatory variables are sorted in decreasing order according to the permutation importance of ODH. The top five variables of each tree species group are highlighted in black.

To avoid unwanted collinearity effects in our model, we eliminated strongly correlated variables. For example, we retained ISF as the only light variable in our dataset. The variables described in the sections “Vegetation surveys” and “Additional Variables” are our explanatory variables in the random forest model. The final selection of explanatory variables is provided in the Figures 2 (Question I and II) and 4 (Question III). Hunting variables, such as roe deer harvest, could not be included for the height growth prediction, as they refer to a hunting district (which may include multiple monitoring areas) and do not impact height growth inside fenced plots.

We fitted a separate random forest model for all tree species groups. The models used to address questions I and II are identical. Furthermore, for research aspect III, we stratified between the two hunting regimes, to differentiate between the “willingness for change” and “no-change” group (see section “Hunting”).

All data manipulation and statistical analysis was executed in the statistical programming language R v. 4.1.2. We applied the R-package randomForest for fitting our random forest models^65^. We determined the variable importance according to Breiman^61^ as permutation importance. To display the marginal effect of explanatory variables on the dependent variable we fitted partial plots, provided by the randomForest R-package (see below). We took in-data variability into account by bootstrapping 85% of our data per partial fit. To identify trends from the resampled predictions, we applied the loess function to create a smoothed tendency curve. Each prediction is based on 500 ‘trees’ with a minimal node size of 5. We based our logistic mixed effect regression on the lme4 package^66^.

## Results

### Variables influencing height growth (Question I and II)

On average, annual height growth varied over all tree species groups between minimal 17.75 cm to 19.15 cm year^−1^. We found in one ODS plot the highest median growth rate of 68 cm year^-1^ and the lowest for ODH with − 34.5 cm year^−1^. A negative growth rate is possible, as we could not track individual trees; a total failure of the saplings appears negative on median between two subsequent years.

The species-separated random forest model could explain  > 20% of variance for deciduous trees (ODH = 22%, ODS = 30%). However, we found a rather low pseudo r-squared for the conifers of 11%. The generally small sample size and the large variation between growth rates between monitoring areas contributed to the low pseudo r-squared of the conifers.

Here, we present the impact and marginal effect of a selected group of coefficients on the height development of the admixed tree species groups. The complete overview of all marginal effects can be found in the supplements (1st PDF).

**Figure 3:**
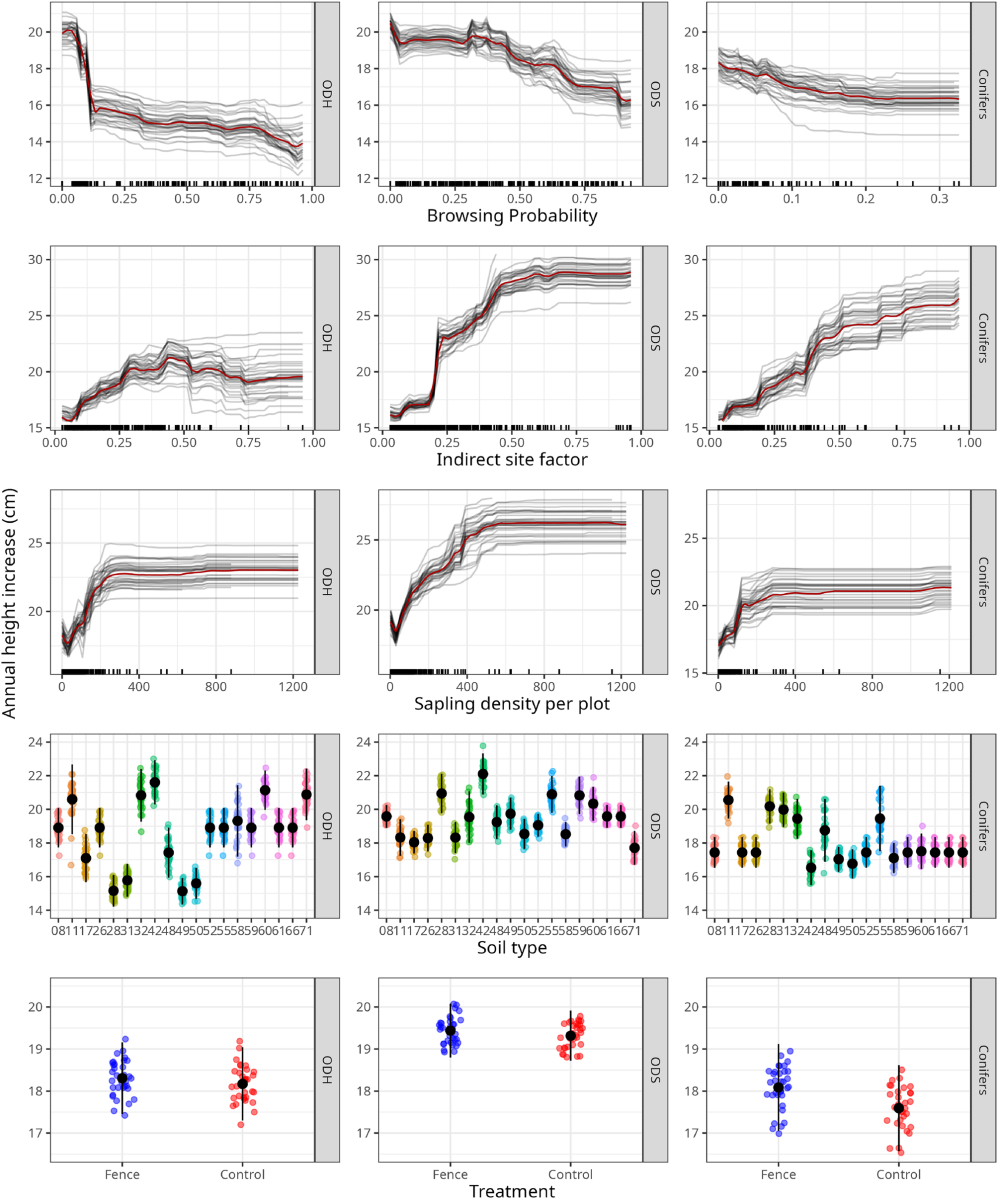
Marginal effects for selected explanatory variables predicting the median annual height increase in the tree species groups ODH (other deciduous tree species, high life expectancy), ODS (other deciduous tree species, short life expectancy) and conifers. A legend for the soil types can be found in the Supplementary Table S3.

At least one *light availability* variable was highly influential for all admixed tree species groups (see Fig. 2). Up to a certain level, light availability was positively correlated with height growth. However, we found the general trend that after a certain threshold level was crossed (e.g. above an indirect site factor of 0.5), height increase stagnated or even slightly decreased (see Fig. 3). That said, our sample size above that threshold was limited. The data distributions (rugs above the x-axis) reveal the focus of our study on “dark” beech dominated forests.

The *number of saplings per plot*, implemented to represent inter- and intraspecific competition, has a medium (conifers and ODS) to high (ODH) permutation importance. For all tree species groups, we observed a clear tendency of increasing height growth with increasing sapling density (see Figs. 2 and 3).

*Browsing probability* (BP) was a highly height-growth-impacting variable for all tree species (see Fig. 2). However, in comparison to the conifers, the deciduous trees were >6 times more impacted by browsing (see section "Regulating ungulate browsing" and Supplementary Fig. S1). For ODH the median annual height increment was the highest on un- or little (BP < 10%) browsed plots. Crossing the 10% BP threshold led to an abrupt drop in height gain. Beyond this threshold, annual height growth did not recover, but decreased constantly with a low slope. Similar observations could be made with ODS, although here the threshold was reached at a BP of about 40% (see Fig. 3).

We can exclude that the observed abrupt height growth setbacks result from overfitting our model; the rugs, describing the data density on the x-axis, neither formed clusters, which would evince a site-dependent “random” effect, nor do they reveal outliers, due to a low sample size (see Fig. 3). Nevertheless, we could not detect such a sudden setback for the conifers, though a considerable and constant decrease in height increment with rising BP until a BP of ca. 8% was detected. After 8% > BP the negative slope flattened a little. For BP > 20% no reliable predictions can be made, due to insufficient observations (see Fig. 3).

The categorical variable *soil type* played a major role when predicting annual height increment for all three tree species groups (see Figure 2). The conifers performed best on nutrient-rich fluvisols (11) and eutric cambisols (32). Furthermore, we detected high growth rates on dry podzolic cambisols (28, 31) and poor dystric cambisols (55). The largest height growth was found for ODH on eutrophic soils with good water storage capabilities, such as fluvisols (11), eutric cambisols/haplic luvisols (32, 42, 60), cumulic anthrosols (71). ODS grew fastest on haplic luvisols (42). However, high growth rates of ODS were also observed on sites with less nutrients, for instance spodo-stagnic, dystric or spodic cambisols (28, 55, 59). In contrast to ODH, the lowest ODS height growth was found on the nutrient-rich fluvisols (11) and cumulic anthrosols (71) at ODS, while ODH grew the slowest on the nutrient deficient podzolic cambisols (28, 31) (see Fig. 3).

The binary *fencing* information (fence/control) had little to no impact on the height growth prediction. Minor, non-significant fencing effects can be observed for all three tree species groups (see Figs. 2 and 3).

The variables that are mentioned exclusively in the supplement show non-evaluable or -interpretable marginal effects on the height growth prediction, despite sometimes high permutation importances. These marginal effects show either flat curves with a high dispersion of the single estimates of the bootstrapped data, e.g., vegetation length and frost days, or the curves are flat with a local overfit of an outlier, e.g., the aspect variable (see 1st supplementary PDF).

### Regulating ungulate browsing (Question III)

For heavily browsed tree species, the previous section showed that browsing is one of the most important variables influencing height growth and thus mortality of young trees. Therefore, our third research question focuses solely on variables that allow regulation of ungulate impact. A complete list of the marginal effects on BP can be found in the supplementary PDFs 2 and 3.

BPs of the control plots with hunting regime A (without hunting customs), were significantly lower than with hunting regime B for all deciduous tree species groups. ODS had an insignificantly higher BP under hunting regime B for a single year (2018). For the rest of the time series, average BP for the conifers was slightly lower under hunting regime B. The highest BP was found for ODH: in 2020 it was about 46% (hunting regime A) and about 58% (hunting regime B). ODS had about 39% BP under regime A and 48% under regime B at the end of the time series. Browsing on conifers was much lower with 8% BP for both hunting regimes in 2020. Despite the large BP differences between deciduous trees and conifers, over time they all reflect the same increasing or decreasing BP tendencies (see Supplementary Fig. S2).

The coefficients predicting BP of the two deciduous groups in hunting regime A explains > 60% (ODH=67%, ODS=63%) of the variability of the data. The pseudo r-squared for hunting regime B predictions are generally lower than A, though sufficient: for ODH we found 48% and for ODS 56%. The pseudo r-squared for the rarely observed conifers is considerably lower: 40% for regime A and 11% for regime B.

**Figure 4:**
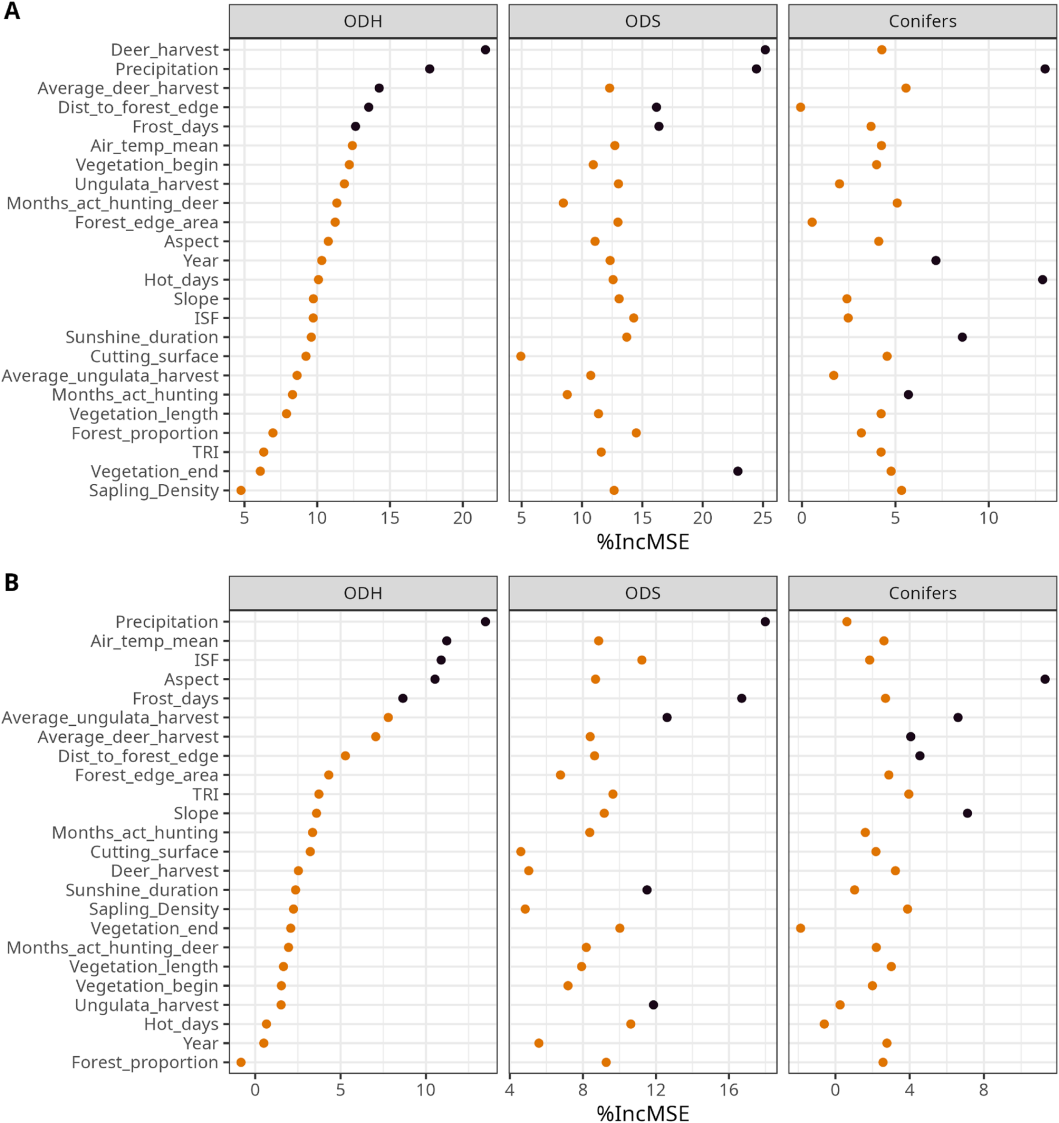
Permutation importance (relative increasing mean square error) predicting BP under hunting regimes A and B for all explanatory variables and the tree species groups ODH (other deciduous tree species, high life expectancy), ODS (other deciduous tree species, short life expectancy) and conifers. The explanatory variables are sorted in decreasing order according to the permutation importance of ODH. The top five variables of each tree species group are highlighted in black.

**Figure 5:**
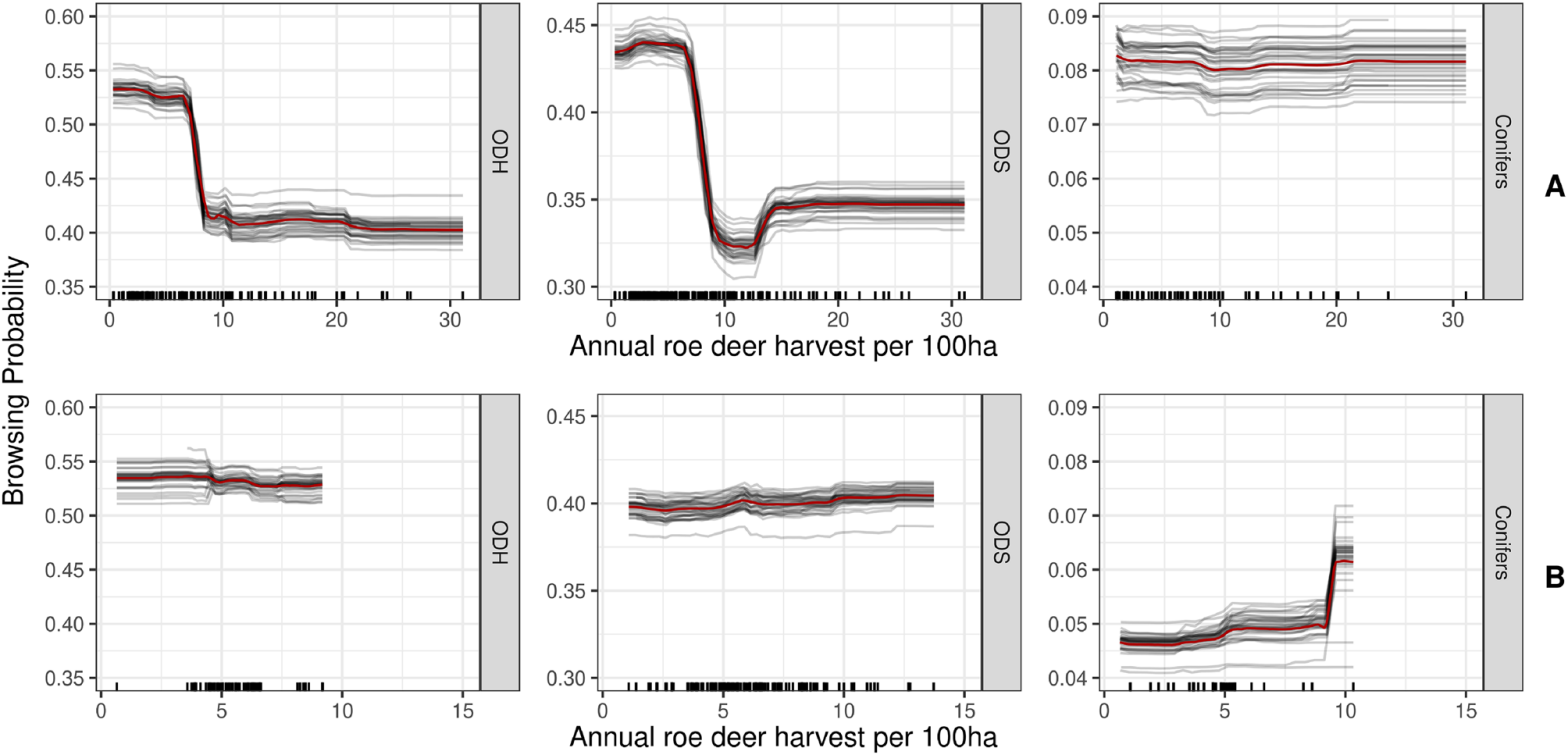
Marginal effects of the annual deer harvest size per 100 ha, predicting the browsing probability (BP), separated in the two hunting regimes A and B and the tree species groups ODH (other deciduous tree species, high life expectancy), ODS (other deciduous tree species, short life expectancy) and conifers.

BP of the two intensively browsed deciduous tree species groups under hunting regime A are both highly impacted by human hunting activities (see Fig. 4A). The *annual deer harvest per 100 ha* is the most important variable, which significantly reduces BP (see Fig. 5A). BP of ODH could be reduced by ca. 15 and ODS by ca. 10 percentage points. It is worth mentioning that a decrease in BP appeared in both species groups at annual harvest levels of around 8 deer per 100 ha, supported by a dense sample density. A tendency of declining BPs can also be explained with the more general ungulate harvest size (see 2nd supplementary PDF). Nevertheless, this variable is not listed among the most BP impacting variables of the deciduous trees.

However, for BP predictions of deciduous trees on sample sites under hunting regime B, hunting variables were not as important as under regime A (see Fig. 4B). Increasing annual deer or ungulate harvest sizes were not correlated with BP (see Fig. 5B). It must, of course, be considered that the hunting regime B has overall lower maximum roe deer harvest rates than A.

## Discussion

We found that competition and light significantly enhance height growth, whereas browsing is detrimental. This negative effect of browsing, however, can be mitigated by sport hunting. Moreover, site factors like soil have a substantial influence on height growth prediction. Therefore, our results support that height growth is the outcome of multiple, interacting factors (Question I)^4,13,31,67,68^. The cited publications discuss individual parameters that determine height growth; our literature research did not find information on a comprehensive parameter ranking. However, Ammer^4^ and Annighofer et al.^31^ provide an insightful perspective by arguing that the effects of these parameters can be masked by heavy browsing. The significant role of browsing by deer on height growth in our study particularly confirms Ammer’s^4^ findings. Complementing this, Walters et al.^67^ also identified interactions between biotic browsing and abiotic environmental drivers. In the subsequent sections, we will delve into a detailed discussion on the primary parameters influencing height growth and their interrelationships as identified through our analyses.

### Browsing impact (Question II)

Our study confirms the findings of other recent studies: that ungulate browsing is an important variable impacting height growth^67,69^. The intensity of browsing by ungulates on a species (group), which indicates their palatability, increases the importance of BP in predicting annual height growth relative to other parameters. Though we could not find direct evidence of sudden drops in height increment after browsing exceeds certain thresholds in the scientific literature, we did locate several previous observations that could support our findings. For instance, Eiberle and Nigg’s^7,36^ critical browsing thresholds were built on the observation that after an average height growth loss of 25%, sapling mortality (of spruce, sycamore maple, silver fir and beech) increases rapidly. Our median height increment losses derived from the marginal effect of BP for the preferentially browsed deciduous saplings ranged between 20% (ODS) and 25% (ODH). However, the thresholds were reached at different BP levels (ODH at ca. 10%, ODS at ca. 40%) and do not match the critical browsing thresholds of Eiberle and Nigg^36^. This might be explained by the predominant branching system of the tree species groups and local light and nutrient availability. ODH includes mostly monopodial growing trees (see Supplementary Table S2), such as sycamore maple, where compensating a terminal shoot is harder, than the sympodial branching ODS group^6,70^. Furthermore, in comparison to ODH, we have more ODS observations at plots that feature more light at the shrub layer (see data distribution of Figure 3). Since, to a certain degree, light availability stimulates height growth, we assume that higher light availability also allows higher levels of BP on forest regeneration before reaching their browsing threshold. The faster the height growth of a sapling, the less likely it is to be (repeatedly) browsed if the BP remains constant over time^71^. Observations by Kupferschmid et al.^29^ also support this thesis, as they found that a higher light availability favors the height growth of browsed and unbrowsed fir saplings in the Swiss alps. Silver and Douglas firs are tree species that are preferentially browsed^37,72^. However, conifer browsing rates are low compared to deciduous trees in our (see Supplementary Fig. S1) and in other studies^32,73^. We explain these relatively low rates by the fact that many deciduous tree species can compensate browsing by developing a Lammas shoot in summer^24,74^. A sapling that dies due to repetitive browsing is no longer included as “browsed” in relative wildlife damage assessments. Fir’s inability to provide Lammas shoots and the overall low model fit might explain why we found a steady decrease in height growth when BP increased, but no clear threshold. Deciduous trees might compensate for browsing, until their vitality and compensatory capacity fail.

It is noticeable that when growth declined after a threshold, some increase remained even at high BP. The reason for this is the experimental design, which only maps living individuals; as long as there are still living individuals in an area, there will also be growth. We also would like to emphasize that we predicted the median height decrease per plot, forecasted across various areas containing both browsed and unbrowsed individuals. Abrupt declines in average height growth have a profound impact on the individual browsed trees, which are the cause of the observed decrease.

BP was only predicted for saplings between 50 and 130 cm. Although Kupferschmid et al.^73^ states that ungulate browsing in Switzerland was most intense in a height class of 41–100 cm, our prediction comes with the risk of an underestimation of ungulate impact, as the saplings below 50 cm are left out^46,75^.

#### Managing the browsing impact (Question III)

Since browsing is a significant factor influencing the height development and thus the overall development of young saplings, it is of great relevance that our results prove that sport hunting mitigate ungulate browsing on selectively browsed deciduous saplings, especially under more stringent hunting regimes (here, regime A). Consultations with forest owners in our study districts confirmed that regeneration tends to be more successful when at least 8 deer per 100 ha were harvested. In contrast, for districts where traditional hunting customs were maintained, we could not detect a browsing mitigation effect. However, we are aware that with our subjective hunting regime classification, we can only speculate as to why deer harvest size has such a large influence on BP under regime A.

Our findings contrast with some past studies based on the harvest-density approach, where the deer harvest size was used as an indicator of ungulate density, positively correlating with browsing impact^62,76,77^. The harvest-density approach is based on the idea that if there are more ungulates in the forest, potentially more will be seen by hunters and therefore more will be shot. In contrast, our approach relies on the fact that with hunting, ungulate densities can be reduced^78^ alongside their browsing impact^46,79^. In the end, the truth probably lies between the two approaches: assuming that ungulates move randomly through the forest and that hunting is executed without selection preferences at randomized locations, harvest size should correspond to the ungulate density as long as it remains below the ungulates’ reproduction rate^76,80^. As soon as the harvest rate exceeds the ungulates’ reproduction rate, ungulate density decreases. Further, Kahlert et al.^77^ stresses that a harvest-population-relationship can only be drawn after an extensive check of the harvest rate validity regarding hunting legislation changes, or the sudden decrease in detectability/accessibility (e.g. habitat changes) of the hunted species.

However, the actual ungulate density for our sample sites is unknown. Thus, we cannot state if the reduced browsing impact on the deciduous species after an annual harvest of ca. 8 deer per 100 ha was due to a reduction of the ungulate density. In general, ungulate densities can only be associated with their browsing impact in the context of the landscape’s carrying capacity^81^. In contrast with cultural carrying capacity, which reflects the (silvicultural) goal of forest stakeholders^82,83^, biological carrying capacity determines the quantity of ungulates an area can sustainably feed. The higher the biological or cultural carrying capacity, the lower the ungulate impact at a constant population size. The significantly reduced, but still high, browsing impact on the preferentially browsed ODH and ODS may indicate that ungulate densities were reduced by hunting below the biological carrying capacity, but are still above the cultural carrying capacity.

All our sample sites are selected based on a high regeneration probability, which favors managed stands with a potentially high light availability^50^. This selection was intentional, since it is precisely in forest gaps that regeneration is to develop successfully. Kuijper et al.^35^ observed in the Białowieża Primeval Forest in Poland that ungulate browsing is a phenomenon clustered in forest gaps. Therefore, our browsing surveys likely reflect the upper end of the BP distribution of our sample sites’ forests, and suggest that there is no linear correlation between ungulate density and browsing impact. In fact, Ramirez et al.^84^ observed that the habitat usage by red, fallow, and roe deer exhibited a non-linear increase in relation to sapling density. Therefore, either a drastic reduction of ungulate density and/or hunting focused on the regeneration sites can reduce browsing in forest gaps^35^. The browsing reduction at higher deer harvests in hunting regime A districts could consequently also support Cromsigt et al.^85^ “hunting for fear” concept, where hunting activities alter how ungulates perceive the risk of becoming prey. The increased hunting intensity that is spatially (risk hotspots), but not temporally predictable for ungulates, distributes ungulate concentration more evenly over the area of the forest or landscape by a predator-like hunting pressure^86,87^. A recent study by Ramirez et al.^43^, also supports our findings regarding the “hunting for fear” concept, as they were able to prove in a camera trap study that deer harvest rates can act as a top-down force, mitigating regeneration patch utilization by deer.

To identify regeneration hotspots and to establish effective hunting regimes there, coordination between silvicultural managers and hunters and a high motivation to hunt is needed^46,85,88^: dense regeneration sites are harder to hunt. Our results show that the communicative principles of the “hunting for fear” concept were implemented in hunting regime A districts: the fact that higher deer harvest sizes mitigated browsing on the preferred deciduous, rather than the less-browsed conifers, is indicative of silvicultural awareness and expertise about the local browsing conditions of the hunters. In other literature, (traditional) hunting or a combination of predation and hunting had either no effect on browsing impact^44^, or only led to a decrease in ungulate browsing on the less preferred, predominant tree species^42,45^. Our observation that the hunting regime B did not generate a substantial effect on browsing is therefore consistent with our hypothesis.

In summary, our stratification by hunting regime underscores that BP reduction is only achievable through higher deer harvest rates if there is a strong motivation to mitigate ungulate impact, as exemplified in our study by hunting regime A. Moreover, it appears that it is not solely the harvest rate per se that alleviates ungulate browsing, but rather the secondary effects that are indicative of these harvest rates, such as the avoidance of heavily hunted regeneration hotspots.

### Light and competition

The beneficial impact of light availability on sapling height growth confirmed the functionality of our model^89,90^. However, as we observed, sapling growth response to higher light availability is limited and the reasons for which are manifold. The shade tolerance of the tree species can impact the height growth saturation point^91,92^. The photosensitive species in the ODH and ODS groups reacted more strongly to an increasing light availability than the conifer group, in which the shade-tolerant silver fir dominates. For instance, shade-tolerant beech saplings only seem to react to an increasing canopy gap size with radial growth^30^, or in the case of silver fir saplings just to a certain threshold of light availability; beyond that threshold annual height growth might stagnate or even decline^27,29^. Furthermore, height growth reactions to light availability are also defined by other factors, such as soil nutrition and water and shrub layer competition^28,67,93^. For instance, it was reported that the height increment of Douglas fir saplings increased linearly with light availability if both soil variables were sufficiently present^28^.

Since increased light availability can not only promote sapling height growth, but also biomass accumulation in the herbaceous and shrub layers^33,34^, we expected that our competition variable “sapling density per plot” would result in lower height growth among the selectively browsed species; the increased biomass occurrence could conceivably increase intra- and interspecific competition in the shrub layer^94^. However, we observed the opposite. On the one hand, light-demanding saplings can have a short-term height-growth-response, which is controlled by a reduced ratio between red and far-red light at high saplings densities^91,95^. On the other hand, our multi-collinearity check found a weak, positive association (ODH: $cor=.21$, ODS: $cor=.18$, conifers: $cor=.35$) between light availability and sapling density. Other studies confirm that large canopy gaps, and therefore a high light availability, result in higher sapling densities; although saturation effects can also be observed in this relation^96,97^.

Nevertheless, we did not consider competition from the ground flora in this study. While Harmer^9^ could not find an effect of ground flora competition on height growth of browsed and unbrowsed saplings in an artificial browsing experiment, it is possible that species such as *Rubus* spp. can negatively interfere with forest regeneration^67^. However, *Rubus* spp. mainly competes for resources in the early stages of forest regeneration; a coexistence next to an established regeneration, as in our case, does not harm the development of forest regeneration^98,99^.

### Soils

The results of the variable soil type have to be considered with caution. On the one hand, they confirm beneficial growth conditions for ODH. The dominant tree species in this category, such as maples, ash and lime trees, prefer deep, fresh, and nutrient-rich soils^13,100^. On the other hand, the versatile species of the ODS group occupy different ecological niches and no general statements can be made. While rowan and birch cope well on nutrient-poor, shallow, and acidic sites, buckthorn (*Rhamnus* spp.) or fly honeysuckle (*Lonicera xylosteum*) require calcareous and nutrient-rich soils^101^. Finally, the categorical variable soil type may not reflect the soil effect on growth conditions for rare soils in our project areas (such as cumulic anthrosols (71)), but rather idiosyncratic features of the site itself with its local browsing and growth conditions—a quasi random effect. For example, the highest (fluvisols (11), dystric cambisols (55)) and lowest (haplic luvisols (42)) growth estimates for the conifers group are found for soil types that are only represented by a few plots (see Supplementary Table S3).

### Fencing

At first glance, the generally low effect of fencing on our height prediction appeared unexpected—particularly given that the exclosures were designed to safeguard sapling growth against the inhibiting effects of browsing. However, to the best of our knowledge, there were no ungulate browsing observations on saplings inside the exclosures that could indicate potential fence-leakages. The minor importance of fencing could be better understood by considering unbrowsed or lightly browsed control plots, which are inadequately represented by the fencing variable. At the same time, the BP variable, which is also included in the model, has the predictive power for such plots and therefore contributes to the low significance of fences.

In addition, the fencing effects could be linked to various unobserved factors apart from browsing: ungulates can reduce shrub layer competition by reducing the abundance of species like *Rubus* spp.^102^ (also see section "Light and competition"); moreover, fenced plots do not benefit from fertilization and seed dispersal by ungulate feces^2^, and are not impacted by changed soil conditions due to ungulate rooting (wild boars)^103^ and trampling^1^. Therefore, the low importance of fencing suggests we achieved our goal of similar conditions in both fenced and control plots.

## Concluding remarks

The height growth of the admixed tree species in our plots is a multifactorial phenomenon. Height growth is an easy-to-measure variable that is sensitive to current and past ungulate impact on selectively-browsed forest regeneration. It is also closely related to sapling mortality. Growth-enhancing bottom-up effects, such as nutrient-rich soils and a sufficient light availability, could support the resilience of browsed saplings to a certain extent. If the browsing impact exceeds a certain threshold, however, the height growth of selectively-browsed tree species drastically collapses. Therefore, we recommend forest owners who care about preserving overbrowsed tree species not to exceed these thresholds. In regularly conducted regeneration inventories, ungulate impact can be measured either by quantifying sapling density or browsing intensity (if local thresholds are known). This information can be an important basis to plan hunting spatially and temporally and to retrospectively monitor hunting efforts. If ungulate impact is to be reduced, we recommend that forest owners and hunters work closely together, e.g., to identify regeneration and therefore hunting hotspots, and to establish motivations to hunt without trophy customs, e.g., by a financial compensation for hunting-induced silvicultural success.

## Supplementary Information

Supplementary Information.

## Data Availability

The datasets used and/or analyzed during the current study are available from the corresponding author on reasonable request.
