# Supplementary material for: Abrupt height growth setbacks show overbrowsing of tree saplings, which can be reduced by raising deer harvest
Source: Sci Rep. 2023 Jul 25;13:12021. doi: 10.1038/s41598-023-38951-8 (PMC10368749; doi:10.1038/s41598-023-38951-8)
Supplement: Supplementary file 1 — Supplementary Information. [file 41598_2023_38951_MOESM1_ESM.zip › Supplementary_Information_File.pdf]

Supplementary Information file for
Abrupt height growth setbacks show overbrowsing of tree saplings, which can be reduced by raising deer harvest

Kai Bödeker[1,*], Claudia Jordan-Fragstein[2], Torsten Vor[3], Christian Ammer[4], and Thomas Knoke[1]

[1]Institute of Forest Management, Department of Life Science Systems, TUM School of Life Sciences Weihenstephan, Technical University of Munich, Hans-Carl-von-Carlowitz-Platz 2, 85354 Freising, Germany
[2]Chair of Forest Protection, Institute of Silviculture and Forest Protection, Tecnical Universeity of Dresden, Pienner Straße 8, 01737 Tharandt, Germany
[3]Faculty of Resource Management, University of Applied Sciences and Arts, Büsgenweg 1a, 37077 Göttingen, Germany
[4]Silviculture and Forest Ecology of the Temperate Zones and Centre for Biodiversity and Sustainable Land Use, University of Göttingen, Büsgenweg 1, 37077 Göttingen, Germany
[*]Corresponding Author; kai.boedeker@tum.de

Supplementary Figures

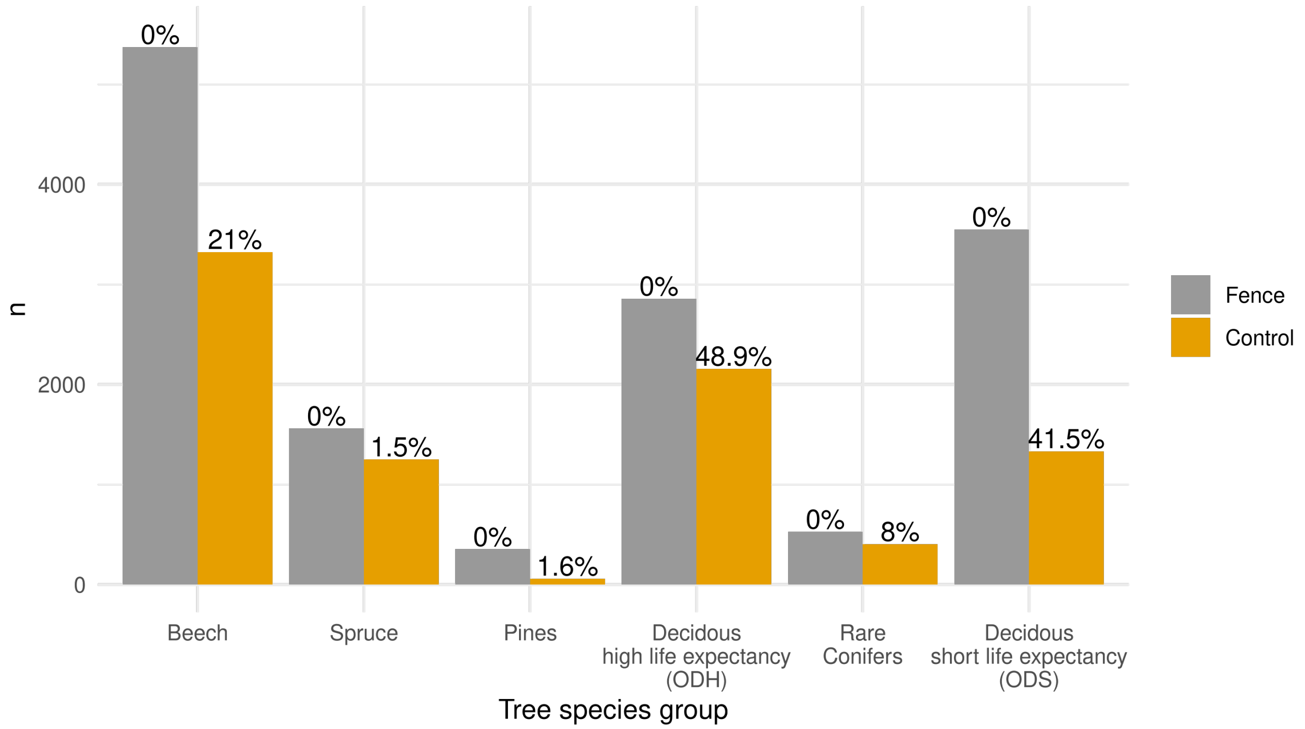

Figure S1. Absolute sapling observations of all tree species groups in 2020, separated by treatment (fence and control) for 2020. Black numbers above the histograms are the average browsing probabilities.

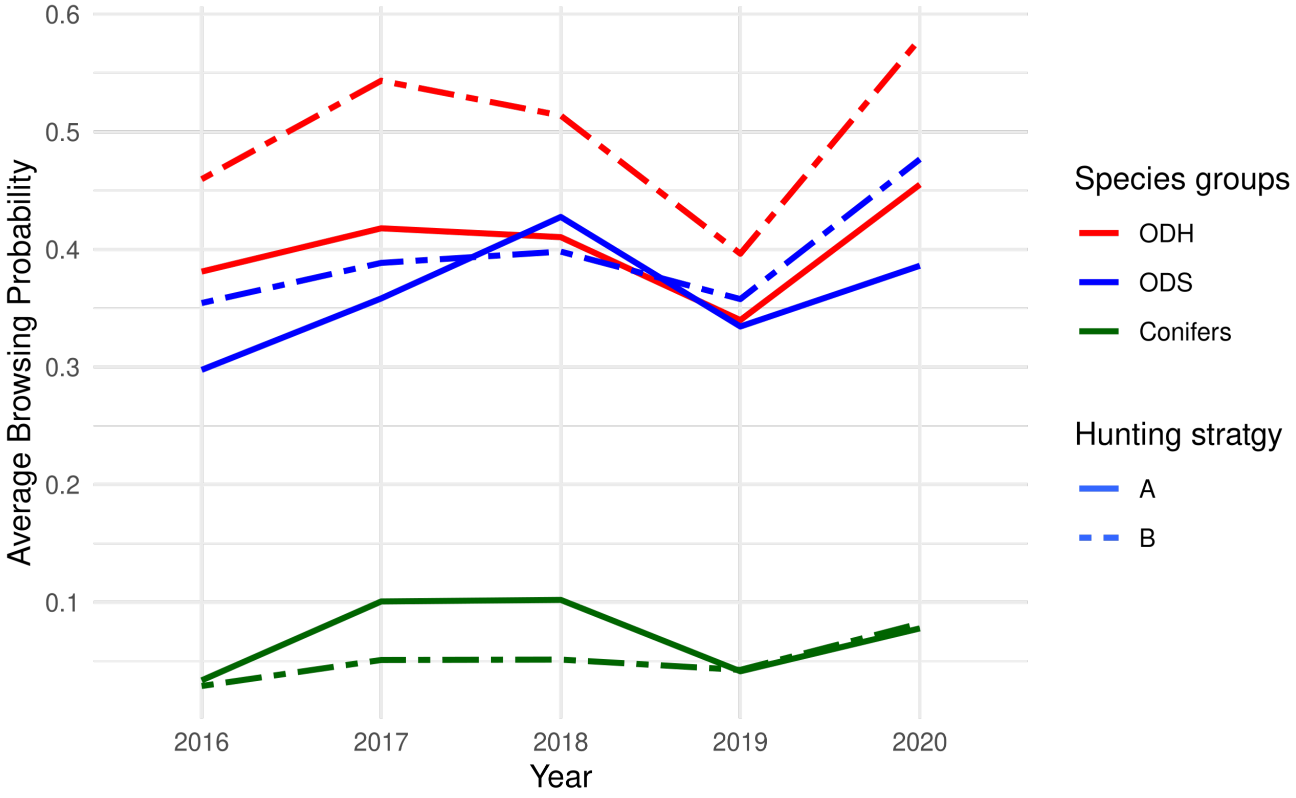

Figure S2. Time series of average browsing probabilities of the control plots of the tree species groups ODH (other deciduous tree species, high life expectancy), ODS (other deciduous tree species, short life expectancy) and conifers; separated in the hunting regimes A and B.

Supplementary Tables

Table S1. Overview of all variables raised within the scope of our project with their respective classes. Factors are used to represent categorical data.

Variable	Class	Unit
State	factor	–
Year	factor	–
Hunting regime	factor	–
Monitoring area	factor	–
Treatment (fence)	factor	–
Plot	factor	–
Genus	factor	–
Species	factor	–
Height	numeric	cm
Initial median height per plot	numeric	cm
Browsing terminal shoot (old)	logical	–
Browsing terminal shoot (fresh)	logical	–
Browsing probability	numeric	–
Sapling Density	integer	$(100\ m)^{-2}$
Roe deer harvest	numeric	$(100\ ha)^{-1}$
Ungulata harvest	numeric	$(100\ ha)^{-1}$
Months of active deer hunting	numeric	month
Months of active hunting	numeric	month
Average deer harvest per active hunting month	numeric	$(100\ ha * month)^{-1}$
Direct site factor (DSF)	numeric	–
Indirect site factor (ISF)	numeric	–
Total site factor (TSF)	numeric	–
Distance to forest edge	numeric	m
Forest circumference	numeric	m
Forest area	numeric	m^2
Forest Proportion around the plot	numeric	–
Annual mean air temperature	integer	°C*10
Days with min. temp. below 0°C	integer	–
Days with max. temp. above 30°C	integer	–
Annual precipitation	integer	$mm * a^{-1}$
Sunshine duration	integer	$h * a^{-1}$
Vegetation begin	integer	–
Vegetation end	integer	–
Vegetation length	integer	day
Elevation	numeric	m
Topographic Position Index (TPI)	numeric	–
Terrain Ruggedness Index (TRI)	numeric	–
Roughness	numeric	–
Slope	numeric	–
Aspect	numeric	°
Soil texture	factor	–
Soil type	factor	–

Table S2. Number of sapling observations per genus of the tree species groups.

ODS		ODH		Conifers	
Genus	n	Genus	n	Genus	n
Sorbus	1948	Acer	1701	Abies	928
Rhamnus	1154	Carpinus	1206	Pseudotsuga	547
Lonicera	1150	Fraxinus	441	NA	NA
Betula	786	Quercus	188	NA	NA
Sambucus	430	Tilia	135	NA	NA
Crataegus	227	Castanea	15	NA	NA
Corylus	220	Robinia	13	NA	NA
Prunus	164	Aesculus	5	NA	NA
Populus	123	NA	NA	NA	NA
Cornus	118	NA	NA	NA	NA
Ribes	100	NA	NA	NA	NA
Alnus	69	NA	NA	NA	NA
Salix	51	NA	NA	NA	NA
Daphne	35	NA	NA	NA	NA
Cytisus	24	NA	NA	NA	NA
Cotoneaster	17	NA	NA	NA	NA
Rosa	13	NA	NA	NA	NA
Viburnum	13	NA	NA	NA	NA
Ligustrum	5	NA	NA	NA	NA
Symphoricarpos	5	NA	NA	NA	NA
Ginkgo	3	NA	NA	NA	NA
Amelanchier	1	NA	NA	NA	NA

Table S3. Number of sapling observations per soil type of the tree species groups.

Soil type number	ODH	ODS	Conifers
11	5	93	5
17	281	229	NA
26	5	331	NA
28	141	1720	93
31	137	300	129
32	608	318	17
42	1674	356	28
48	34	274	139
49	205	678	13
50	173	1123	51
52	NA	5	NA
55	NA	49	5
58	31	351	946
59	NA	12	NA
60	394	556	49
66	NA	2	NA
71	16	259	NA

Supplementary PDFs

Supplement_1_Height_Prediction_partial_plots.pdf

Overview of all marginal effects affecting the height prediction for all three tree species groups (ODH (other deciduous tree species, high life expectancy), ODS (other deciduous tree species, short life expectancy) and conifers).

Supplement_2_Browsing_Prediction_A_partial_plots.pdf

Overview of all marginal effects affecting the browsing probability for all three tree species groups (ODH (other deciduous tree species, high life expectancy), ODS (other deciduous tree species, short life expectancy) and conifers) with the hunting regime A.

Supplement_3_Browsing_Prediction_B_partial_plots.pdf

Overview of all marginal effects affecting the browsing probability for all three tree species groups (ODH (other deciduous tree species, high life expectancy), ODS (other deciduous tree species, short life expectancy) and conifers) with the hunting regime B.
