# Supplementary figures and images for: Abrupt height growth setbacks show overbrowsing of tree saplings, which can be reduced by raising deer harvest
Source: Sci Rep. 2023 Jul 25;13:12021. doi: 10.1038/s41598-023-38951-8 (PMC10368749; doi:10.1038/s41598-023-38951-8)

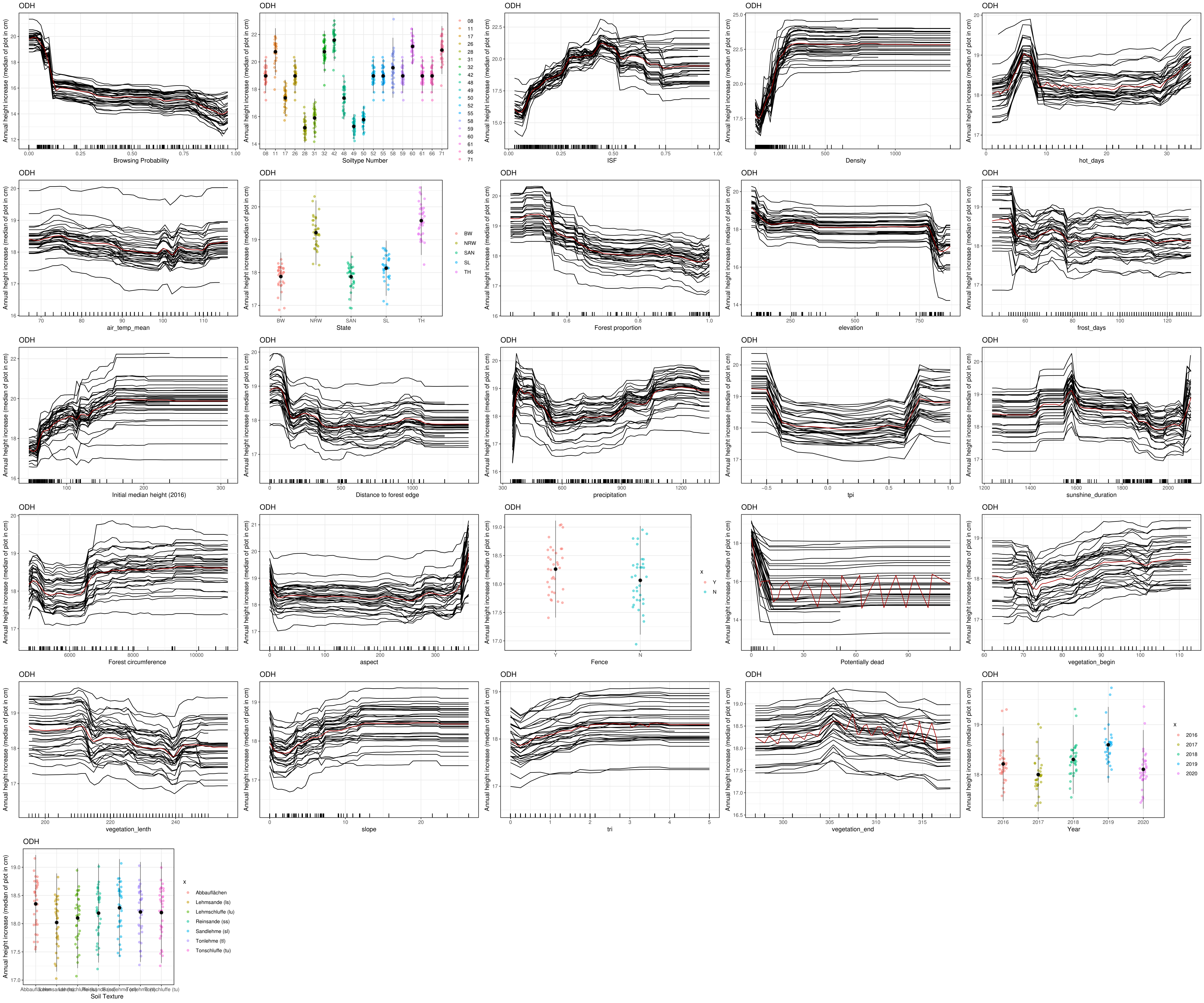

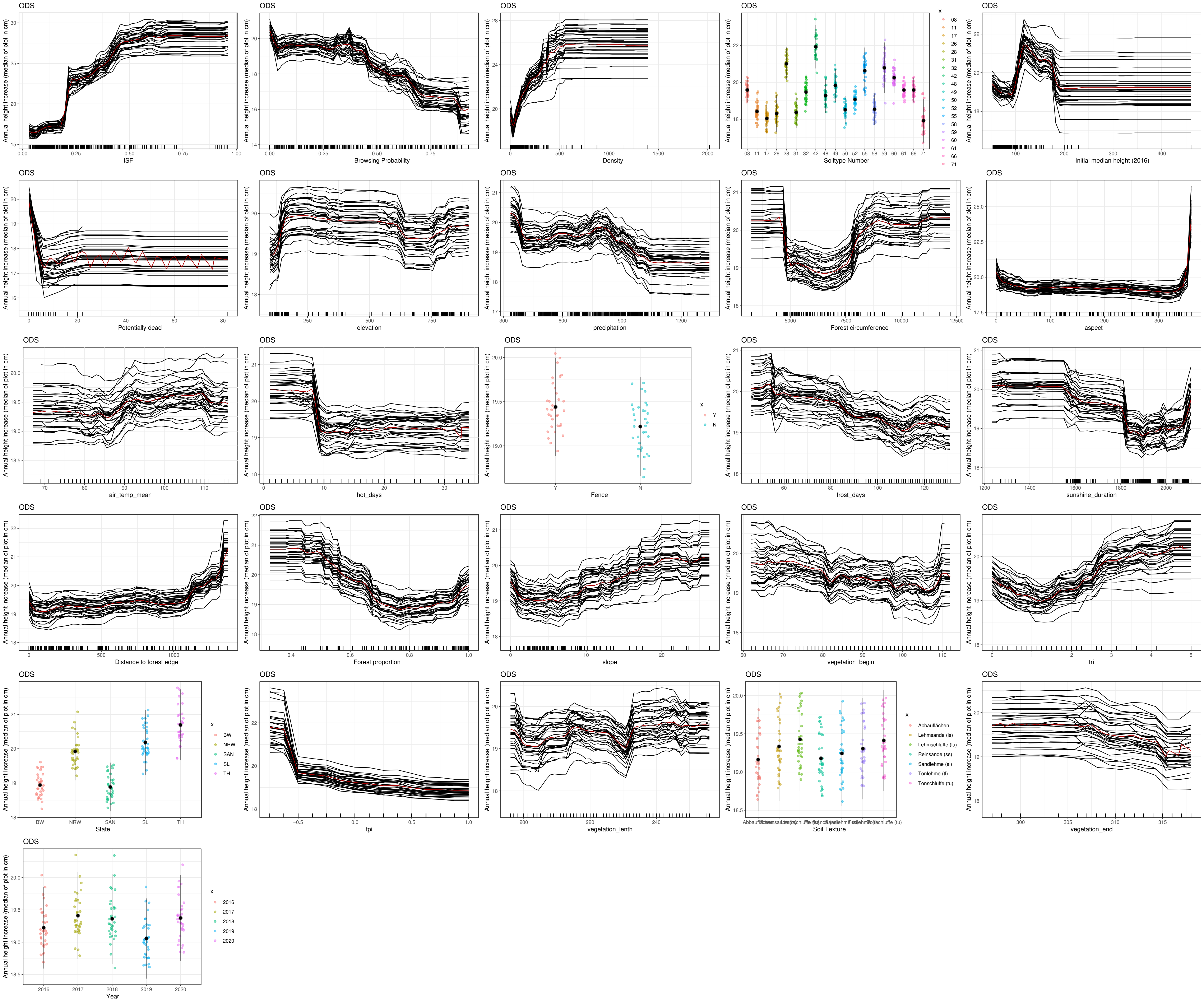

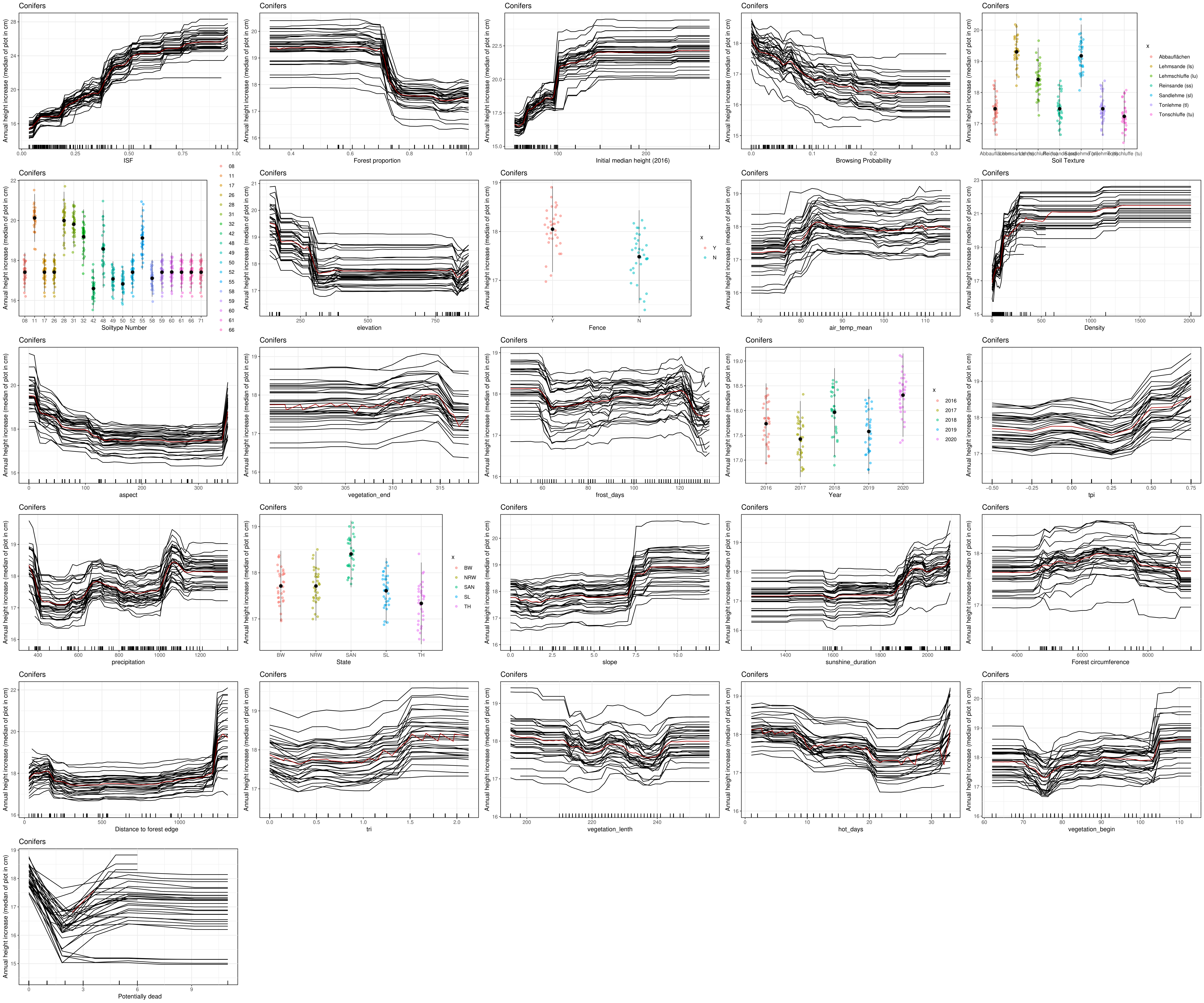

Supplement: Supplementary file 1 — Supplementary Information. [file 41598_2023_38951_MOESM1_ESM.zip › Supplement_1_Height_Prediction_partial_plots.pdf]

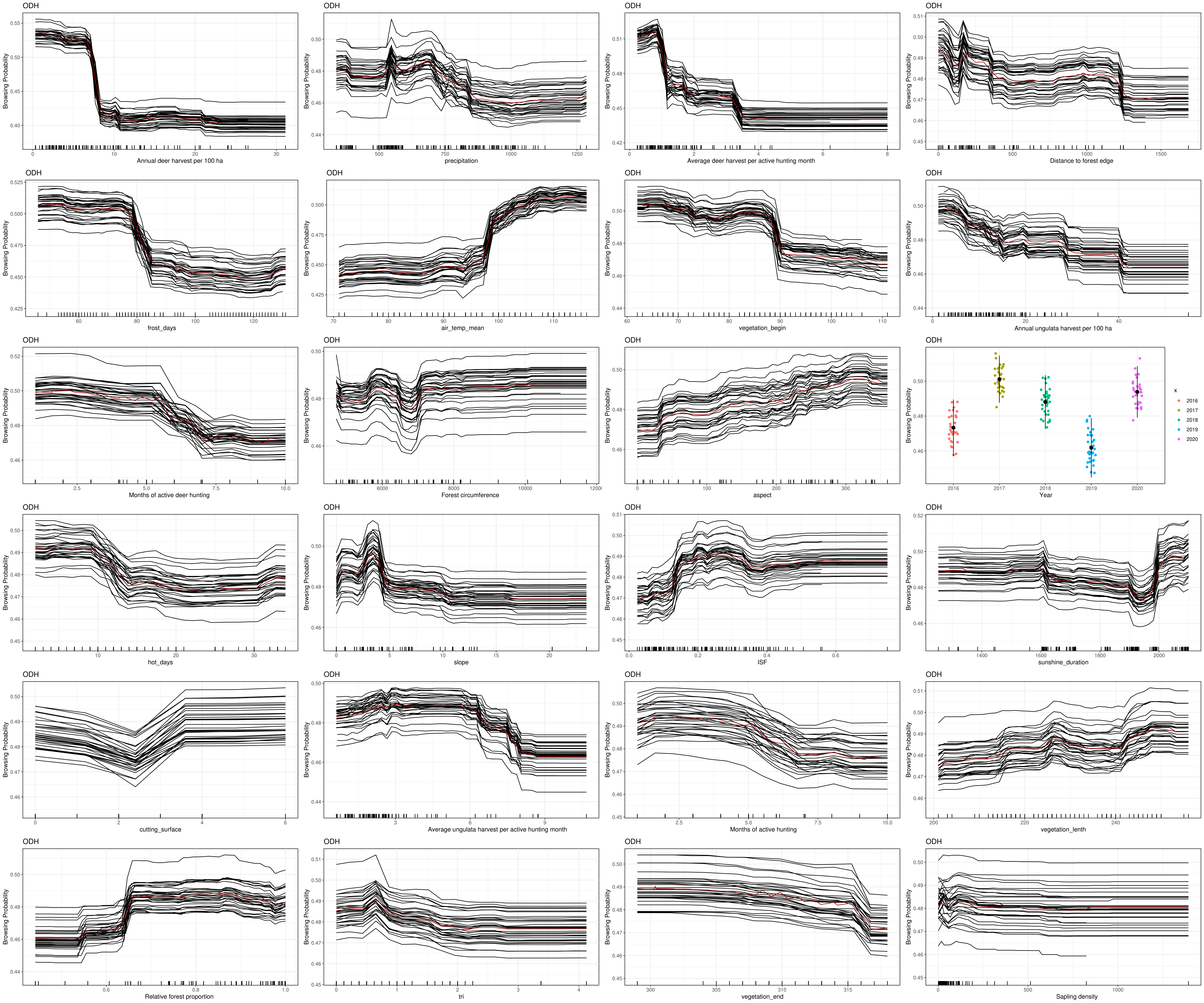

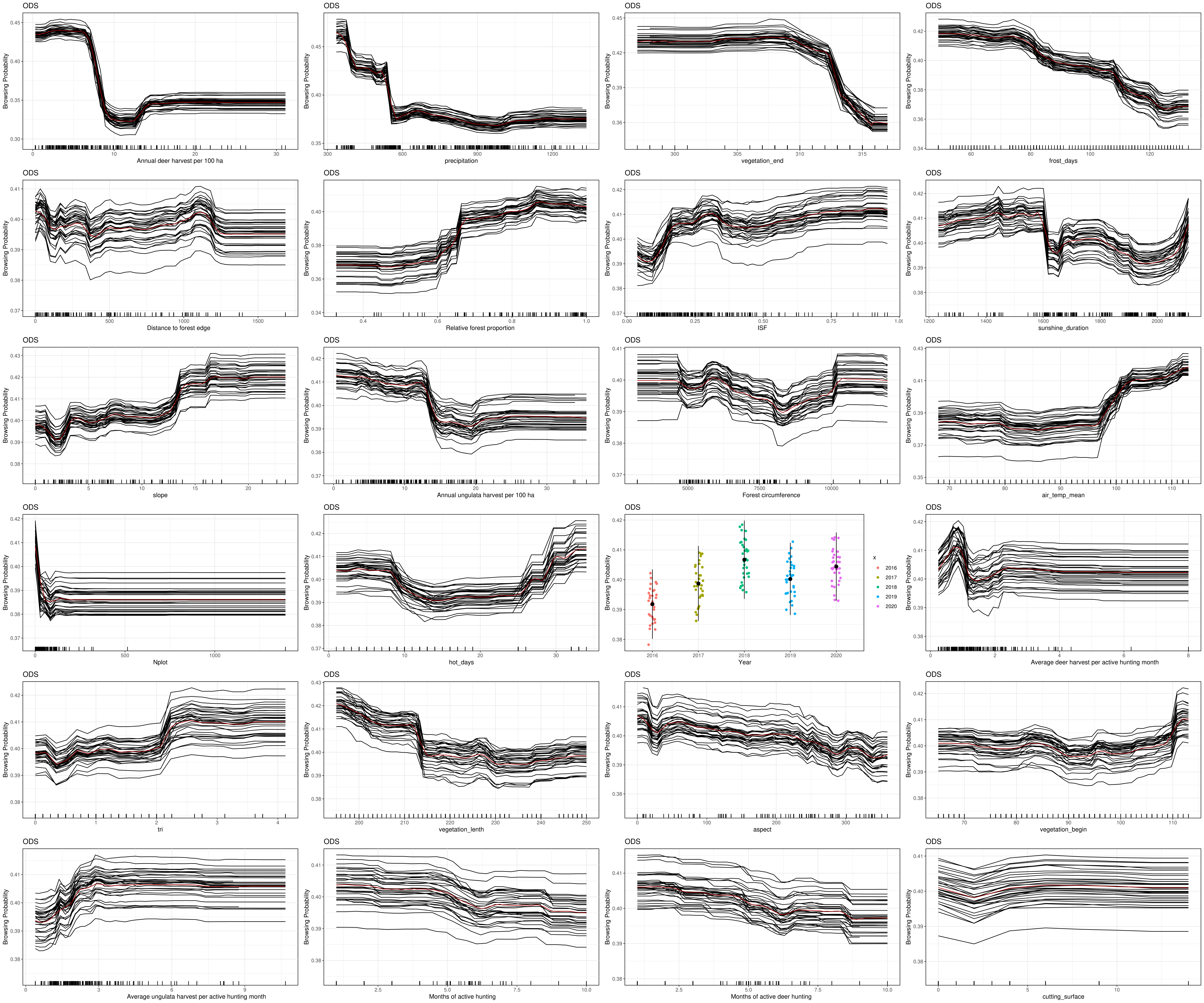

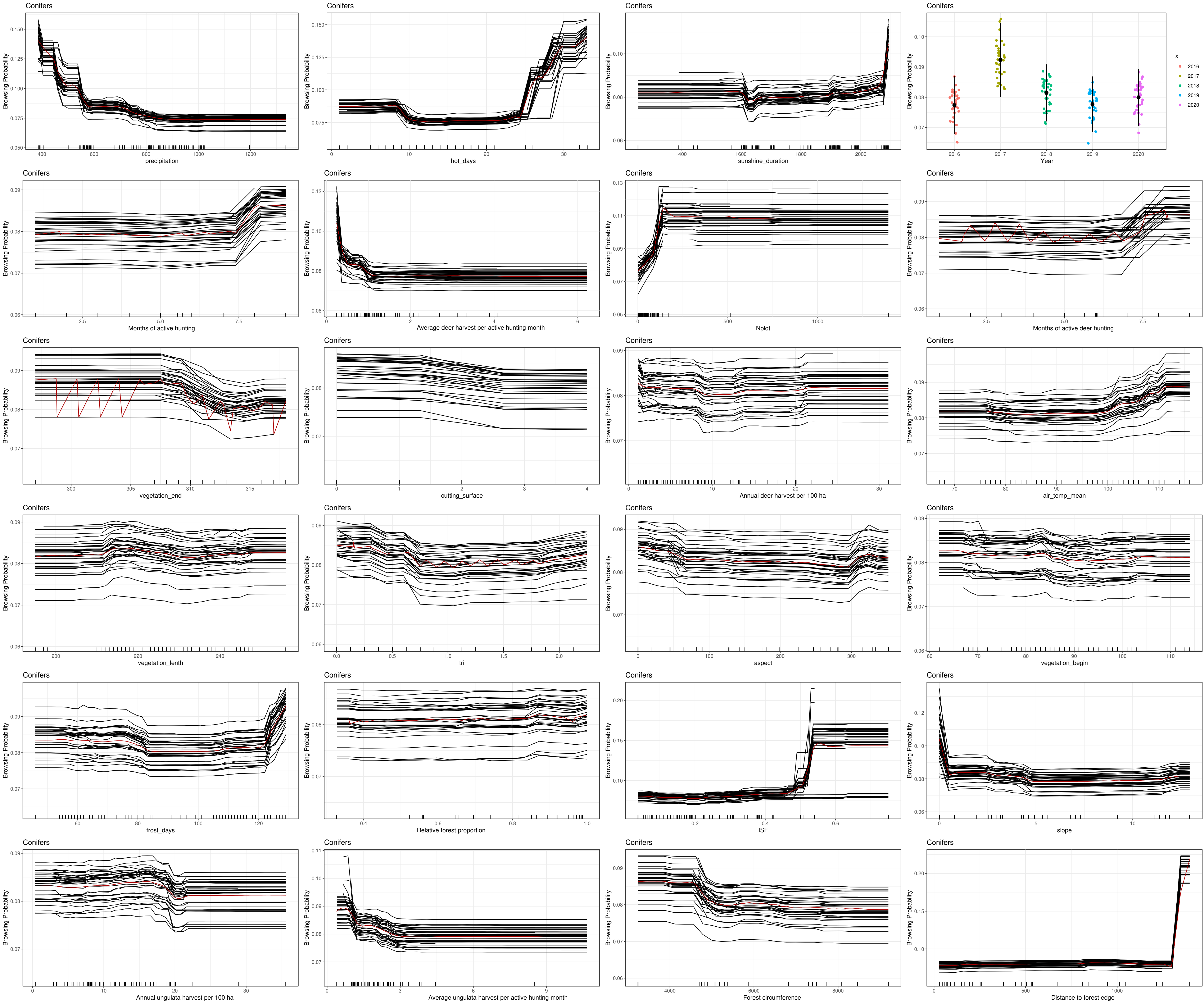

Supplement: Supplementary file 1 — Supplementary Information. [file 41598_2023_38951_MOESM1_ESM.zip › Supplement_2_Browsing_Prediction_A_partial_plots.pdf]

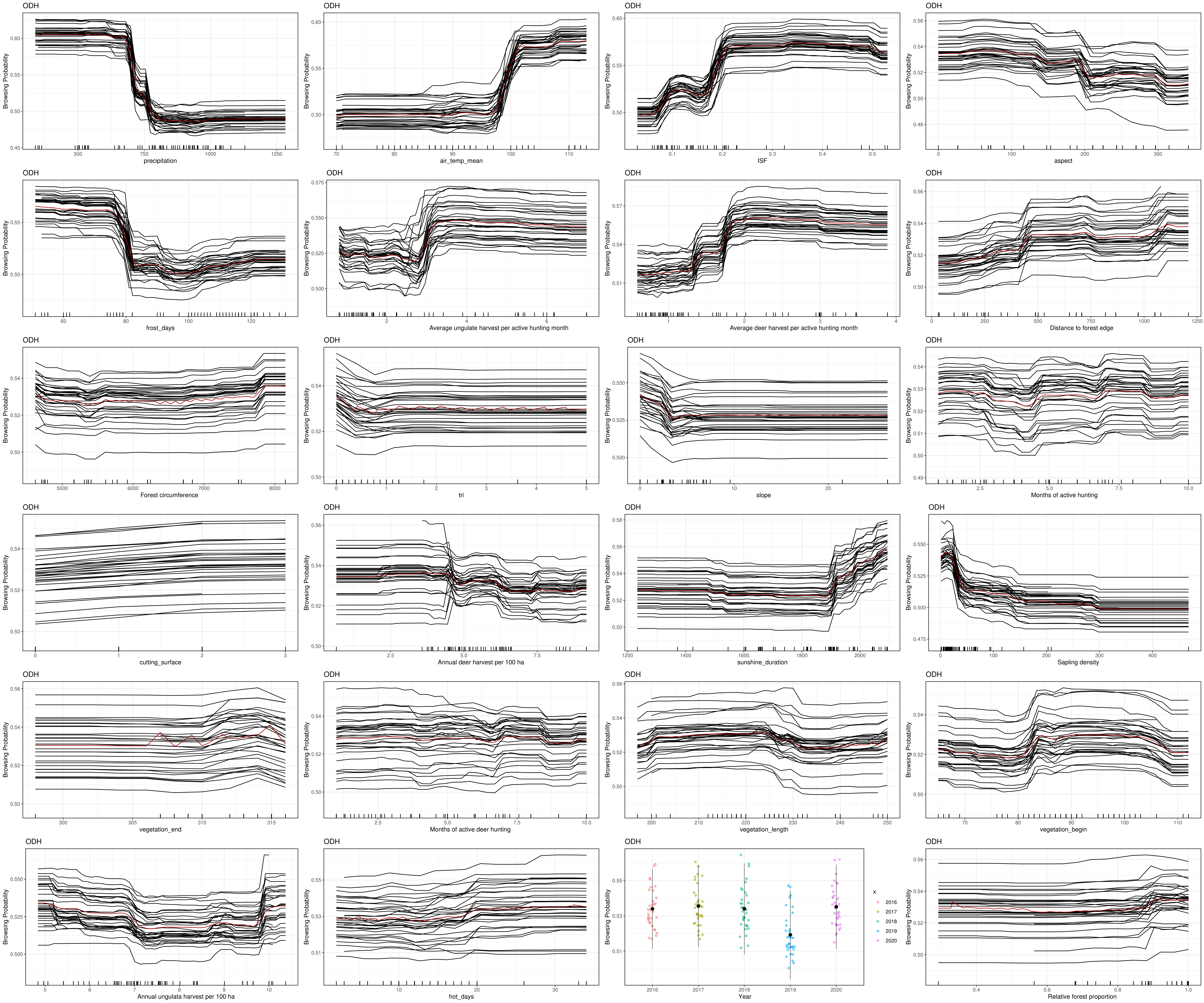

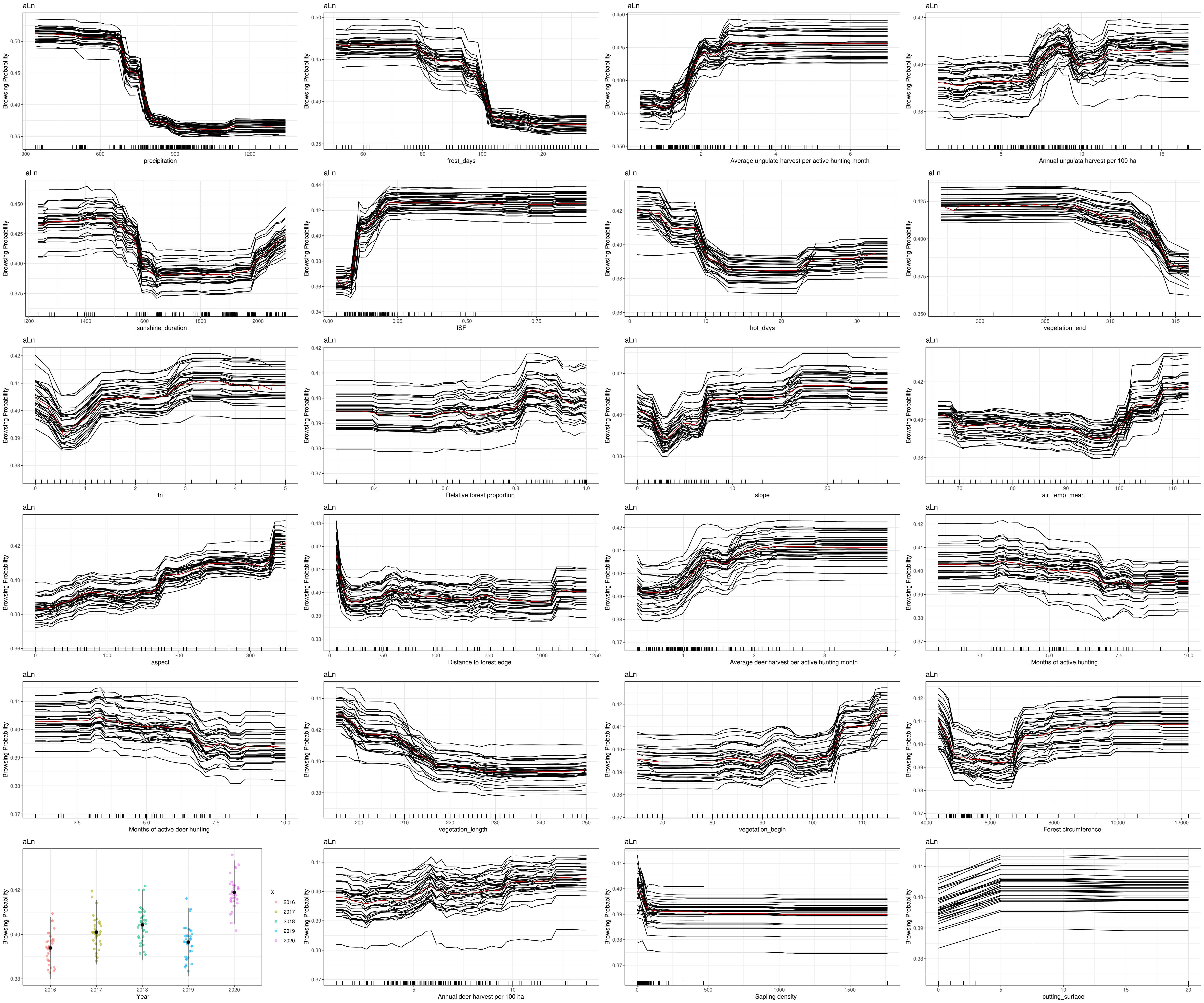

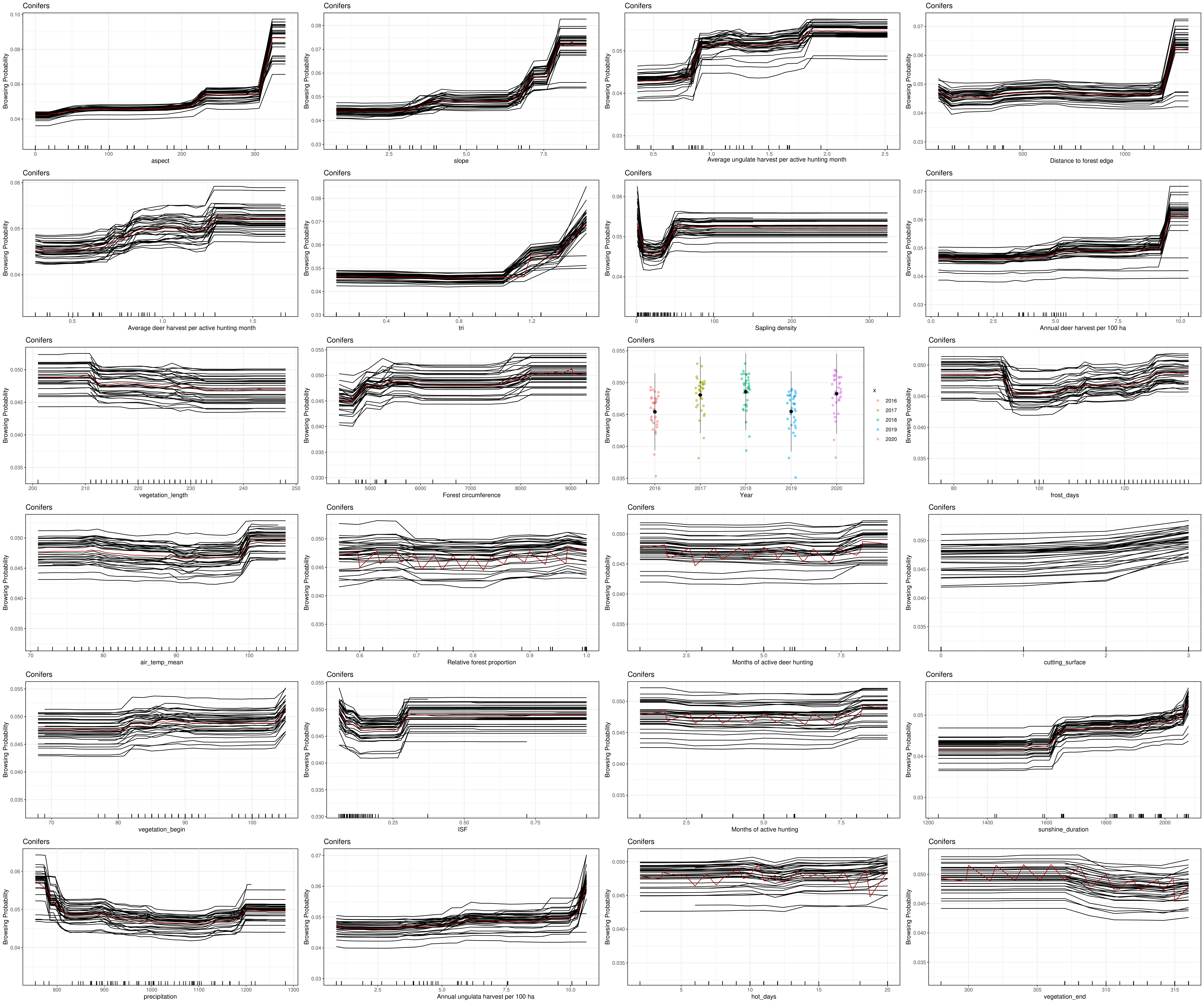

Supplement: Supplementary file 1 — Supplementary Information. [file 41598_2023_38951_MOESM1_ESM.zip › Supplement_3_Browsing_Prediction_B_partial_plots.pdf]
